# Evaluation of microbiological cultures in patients with acute cholecystitis

**DOI:** 10.3389/fsurg.2026.1739629

**Published:** 2026-03-16

**Authors:** Dániel Váczi, Mária Matuz, Ria Benkő, Illés Tóth, Emil Terhes, Edit Hajdú, Erika Papfalvi, András Nagy, György Lázár, Szabolcs Ábrahám

**Affiliations:** 1 Department of Surgery, University of Szeged, Szeged, Hungary; 2 Institute of Clinical Pharmacy, University of Szeged, Szeged, Hungary; 3 Emergency Department, University of Szeged, Szeged, Hungary; 4 Infectious Disease Ward, Department of Internal Medicine, University of Szeged, Szeged, Hungary; 5 Department of Radiology, University of Szeged, Szeged, Hungary

**Keywords:** acute cholecystitis, anti-bacterial agents, bacterial, bile, cholecystectomy, drug resistance, microbiology

## Abstract

**Background and aims:**

Acute cholecystitis (AC) requires emergency care. Besides surgical interventions, adequate empirical antibiotic therapy is indispensable. We aimed to analyze bile samples obtained during surgical interventions for AC. The primary aim was determine sampling frequency, pathogen prevalence (including ESKAPE strains), and to create the cumulative antibiogram of the most common bacteria (including MDR pathogens). A secondary aim was to explore the potential relationship between pathogen type (ESKAPE, MDR) and different patient characteristics in acute cholecystitis.

**Methods:**

A retrospective observational study was conducted. Bile samples from patients undergoing acute cholecystectomy or percutaneous transhepatic gallbladder drainage for acute cholecystitis between 2005 and 2019 were analyzed. Cases were retrieved based on the respective ICD-10 codes. Descriptive and univariate methods were used.

**Results:**

During the study period there were 656 patients with AC; bile samples for microbiology were collected in 379 cases (57.8%). Overall, 412 bacteria, predominantly Gram-negative microbes (60.9%), were isolated. The most common bacteria included *Escherichia* spp. (25.7%), *Streptococcus* spp. (13.8%), *Enterococcus* spp. (13.6%). The proportion of MDR strains was 14.9%. Meanwhile, 109 of 412 pathogens (26.46%) were ESKAPE pathogens. A higher grade of inflammation was associated with a higher incidence of ESKAPE pathogens. *E. coli* exhibited high susceptibility (>90%) to third- and fourth-generation cephalosporins and carbapenems, but lower susceptibility to ciprofloxacin (80%) and sulfamethoxazole–trimethoprim (78%), with 25% of isolates being MDR. Among Gram-positive bacteria, 14.3% of Enterococcus spp. were vancomycin-resistant, while no MRSA was detected in bile samples.

**Conclusion:**

Microbiological sampling, identifying the most common pathogens and determining the antibiotic resistance profile in AC is important to determine the optimal empirical antibiotic choice.

## Introduction

Acute cholecystitis (AC) is one of the most common conditions requiring emergency care. The most common cause of acute cholecystitis (AC) is acute calculous cholecystitis (ACC). The prevalence of gallstones ranges from 10% to 15% in the general population (1). In patients with gallstones, the lifetime prevalence of acute calculous cholecystitis is up to 12% (2, 3). According to the Tokyo Guidelines classification published in 2013 and 2018 (TG13/18), the grade of inflammation in AC is significantly correlated with 30-day mortality (grades 1, 2, and 3: 2.4%, 4.7%, and 8.4%, respectively) (4). Adequate antibiotic and fluid therapy and, if required, surgery or percutaneous transhepatic gallbladder drainage is indispensable for the treatment of AC.

According to TG18, early cholecystectomy (CCY) is recommended in cases of mild (grade 1) inflammation and wherein the patient's performance status based on the Charlson Comorbidity Index (CCI) and the American Society of Anesthesiologists Physical Status (ASA-PS) classification is ≤5 and ≤2, respectively. Early CCY is also recommended in cases of moderate (grade 2) inflammation and if a patient's CCI is ≤5, ASA-PS is ≤2, and the procedure is to be performed by an experienced surgeon in a large surgery center. If CCI and ASA-PS both exceed the cut-off values the patient is ineligible for early surgery percutaneous transhepatic gallbladder drainage (PTGBD) is recommended in grade 2 and 3. In cases of severe (grade 3) inflammation and if the patient is ineligible for early surgery based on their CCI and ASA-PS, PTGBD is recommended (5).

The Tokyo Guidelines (TG18) recommend routine microbiological sampling and resistance testing during cholecystectomy, however, studies focusing on this aspect have been conducted in very limited numbers.

In the initial and mild stages (grade 1) of AC, the bile is sterile but becomes infected as AC progresses (6). Pathogens can be isolated in 29%–54% of bile samples obtained in patients with AC (7). Accordingly, microbiological sampling during acute CCY and/or PTGBD is also recommended by TG18 to identify pathogens and multidrug-resistant (MDR) strains (8). However, since the turnaround time of microbiological evaluations and antibiotic sensitivity determination may last up to 2–3 days, TG18 recommends starting empirical antimicrobial therapy at diagnosis in all grades (7). The primary role of empirical antimicrobial therapy in AC is to prevent systematic progression, reduce the frequency of surgical site infections, and minimize the risk of treatment failure due to antimicrobial resistance (9).

In clinical practice, special attention should be paid to the ESKAPE group. Rice introduced the ESKAPE acronym in for the group of pathogens capable of “escaping” the biocidal action of antibiotics namely E: *Enterococcus faecium*, S: *Staphylococcus aureus*, K: *Klebsiella pneumoniae*, A: *Acinetobacter baumannii*, P: *Pseudomonas aeruginosa*, E: *Enterobacter spp* (10, 11). Due to antimicrobial resistance among these pathogens, infections with ESKAPE bacteria is associated with significantly higher morbidity and mortality rates as well as higher hospital expenses (10). Therefore it is important to identify at the local hospital level the most common pathogens, their resistance pattern (including the rate of multiresistant bacteria) in order to be able to set up an empirical antibiotic guideline.

As MDR (multidrug-resistant) bacteria become more prevalent in microbiological samples, treatment options for these infections become increasingly limited (12). This highlights the critical need for ongoing surveillance of local antibiotic resistance patterns (7).

Continuous monitoring and updating of the local cumulative antibiogram as well as regular revisions of empirical antibiotic regimens are essential (13, 14). We aimed to analyze bile samples collected during surgical interventions for AC.

This is the first study from Central Europe providing a comprehensive analysis of bile cultures and corresponding cumulative antibiograms in AC patients.

As a secondary objective the relationship between microbiological findings (e.g., ESKAPE, MDR) and grade, indication, time frame, and clinical outcomes of AC were assessed.

## Methods

### Study design and setting

This retrospective single-center study was conducted at the Department of Surgery, University of Szeged. Microbiological results of bile samples collected from patients with acute cholecystitis (AC) during laparoscopic or open cholecystectomy (CCY) or percutaneous transhepatic gallbladder drainage (PTGBD) procedures between 2005 and 2019 were analyzed. During the study period, a total of 656 patients with acute cholecystitis were screened, of whom 242 met the inclusion criteria were included in the final analysis. The study protocol was approved by the Regional Human Biomedical Research Ethics Committee of the University of Szeged (approval number: 81/2020-SZTE).

### Patient selection and data collection

Patients were identified using the institutional electronic hospital information system (MedSolution) based on ICD codes corresponding to AC (K80.0, K80.1, K80.4, K80.6, K81.0, K81.2, K81.9). Only patients undergoing acute CCY and/or PTGBD with intraoperative or interventional bile samples were considered.

The following clinical and demographic parameters were collected:

- age and sex
- comorbidity burden using the Charlson Comorbidity Index (CCI) (47)
- perioperative risk assessed by the American Society of Anesthesiologists Physical Status (ASA PS) classification (46)
- severity of AC (Grade I–III) according to the Tokyo Guidelines 2018 (TG18) (48)
- indication for intervention: acute calculous cholecystitis (ACC), acute acalculous cholecystitis (AAC), empyema vesicae felleae (EVF), hydrops vesicae felleae (HVF), or gallbladder perforation (GP)
- times from symptom onset to hospital admission (≤72 h vs. >72 h)
- in-hospital mortality
- microbiological culture results

### Microbiological methods

Bile samples were collected under sterile conditions during CCY or PTGBD procedures. Samples were cultured on blood agar, MacConkey agar, and anaerobic culture media. Bacterial species identification was performed using MALDI-TOF mass spectrometry. Antibiotic susceptibility testing was carried out using disk diffusion and E-test methods, according to the European Committee on Antimicrobial Susceptibility Testing (EUCAST) guidelines were valid at the time of testing (49).

### Definitions

The ESKAPE group included *Enterococcus faecium*, *Staphylococcus aureus*, *Klebsiella pneumoniae*, *Acinetobacter baumannii*, *Pseudomonas aeruginosa*, and *Enterobacter* spp.

Multidrug-resistant (MDR) bacteria were defined as isolates resistant to three or more antimicrobial classes.

Bile samples were considered sterile when no bacterial growth was detected.

### Exclusion criteria

Patients were excluded if:

- no microbiological sample was obtained,
- sampling occurred more than 3 days after the intervention,
- cultures showed no bacterial growth,
- contamination was suspected (e.g., polymicrobial growth without a dominant pathogen), or
- fungal organisms were isolated.

**Figure 1:**
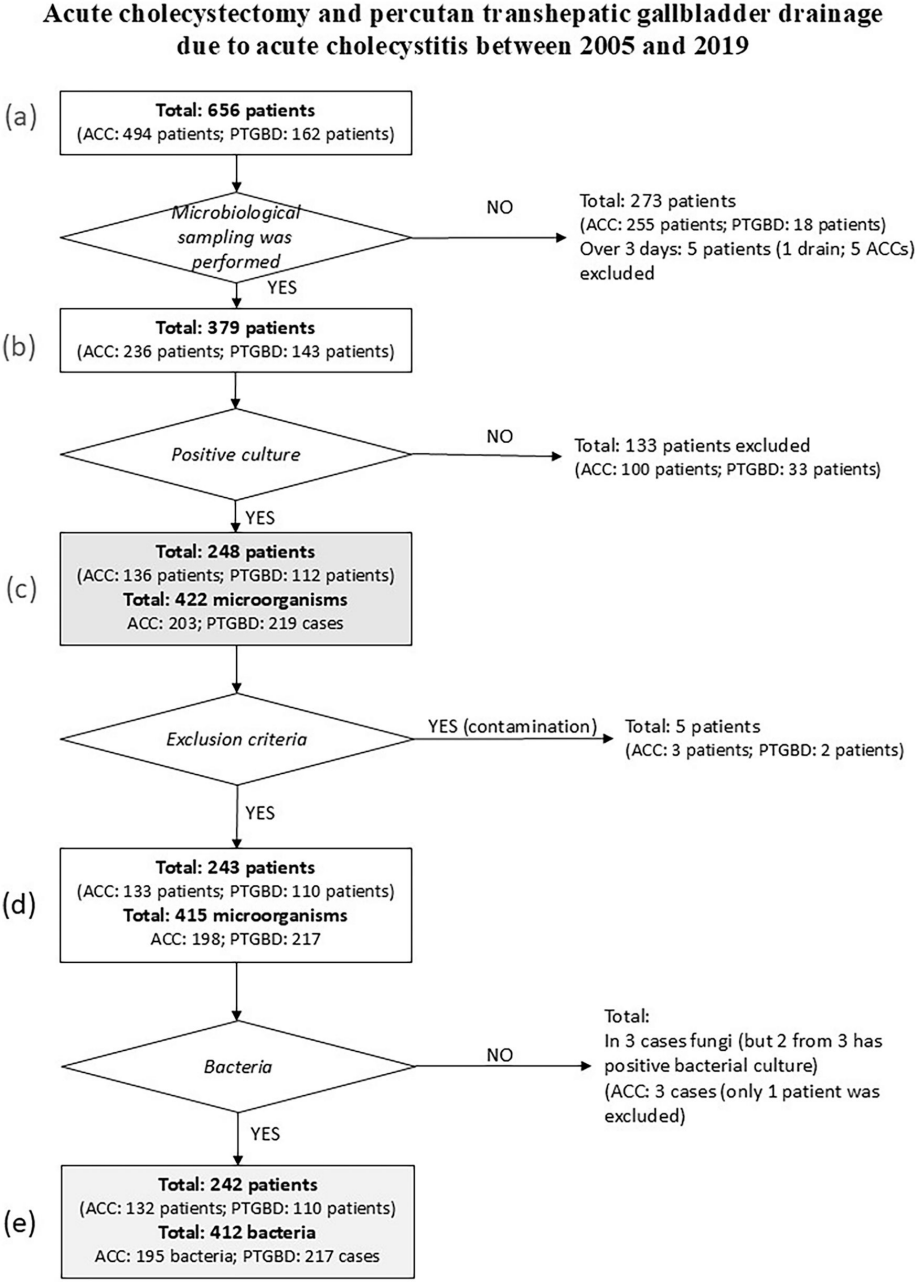
Flowchart of the inclusion and exclusion criteria. ACC, acute cholecystectomy; drain; PTGBD, percutaneous transhepatic gallbladder drainage.

A detailed flowchart is shown in Figure 1.

### Data analysis and outcomes

Patients were categorized into CCY and PTGBD groups. The incidence and distribution of the bacterial isolates were analyzed according to Gram-stain characteristics, ESKAPE classification, and MDR status. Associations between ESKAPE/MDR pathogens and patient characteristics (age, sex, CCI, ASA PS, AC severity, indication, time frame, and mortality) were evaluated. The occurrence of ESKAPE and MDR pathogens was further analyzed according to the AC severity grade and time from symptom onset.

For patients with positive bile cultures, cumulative antibiograms were generated using the MedBakter software, following EUCAST recommendations.

This analysis reflects the local epidemiology and antibiotic resistance patterns of a single tertiary-care surgical center.

### Statistical analysis

Descriptive statistics were used to summarize patient characteristics and microbiological findings. Chi-square tests were applied for categorical variables, and *t*-tests for continuous variables. Univariate analyses were performed to explore associations between pathogen categories and clinical variables. Due to the retrospective design and sample size, only univariate analyses were performed.

Statistical significance was defined as *p* < 0.05. All analyses were conducted using SPSS software.

## Results

During the study period, 656 patients underwent surgical intervention. Acute CCY and PTGBD were performed in 494 and 162 patients, respectively. The sampling ratio gradually increased during the study period. Previously, the sampling ratio was between 26% and 56% only, but it reached 60%–80% during the last 3 years of the study.

### General patient characteristics

Overall, 116 of the 242 patients (47.9%) were men; 61.6% of the patients were aged >65 years. The most common indications for surgical intervention were acute calculous cholecystitis (ACC, 120 patients, 49.6%) and gallbladder perforation (GP, 61 patients, 25.2%). Regarding inflammation, grades 1, 2, and 3 were noted in 53 (22.1%), 157 (65.4%), and 30 (12.1%) patients, respectively. Furthermore, 132 patients (62.3%) were diagnosed with AC within 72 h after the onset of symptoms, 221 patients (93.6%) survived, and 15 patients (6.4%) died (Table 1).

**Table 1:** Patients’ clinical and microbiological characteristics by type of surgical intervention.

Total		CCY	PTGBD	Total	*p*
		132	110	242	
Age	19–65	65 (49.2%)	28 (25.5%)	93 (38.4%)	0.0002
	65+	67 (50.8%)	82 (74.5%)	149 (61.6%)	
Sex	Male	58 (43.9%)	58 (52.7%)	116 (47.9%)	0.197
	Female	74 (56.1%)	52 (47.3%)	126 (52.1%)	
CCI	CCI group 1 (0)	26 (19.7%)	7 (6.4%)	33 (13.6%)	0.0005
	CCI group 2 (1–3)	59 (44.7%)	32 (29.1%)	91 (37.6%)	
	CCI group 3 (4–11)	47 (35.6%)	71 (64.5%)	118 (48.8%)	
ASA-PS	No data	0%	3 (2.7%)	3 (1.2%)	0.001
	1	34 (25.8%)	10 (9.1%)	44 (18.2%)	
	2	51 (38.6%)	41 (37.3%)	92 (38%)	
	3	39 (29.5%)	39 (35.5%)	78 (32.2%)	
	4	8 (6.1%)	17 (15.5%)	25 (10.3%)	
Grade	1	45 (34.4%)	8 (7.3%)	53 (22.1%)	>0.0001
	2	78 (59.5%)	79 (72.5%)	157 (65.4%)	
	3	8 (6.1%)	22 (20.2%)	30 (12.5%)	
	No data	1	1	2	
Indication	AAC	6 (4.5%)	7 (6.4%)	13 (5.4%)	0.001
	ACC	80 (60.6%)	40 (36.4%)	120 (49.6%)	
	EVF	4 (3%)	12 (10.9%)	16 (6.6%)	
	HVF	11 (8.3%)	21 (19.1%)	32 (13.2%)	
	GP	31 (23.5%)	30 (27.3%)	61 (25.2%)	
Time frame	Within 72 h	96 (80.7%)	36 (38.7%)	132 (62.3%)	>0.0001
	after 72 h	23 (19.3%)	57 (61.3%)	80 (37.7%)	
	No data	13	17	30	
Outcome	Cure	129 (97.7%)	92 (88.5%)	221 (93.6%)	0.004
	Exit	3 (2.3%)	12 (11.5%)	15 (6.4%)	
	No data	0	6	6	
GRAM stain	Gram positive	45 (34.1%)	22 (20%)	67 (27.7%)	0.001
	Gram negative	66 (50%)	49 (44.5%)	115 (47.5%)	
	Gram neg. and pos.	21 (15.9%)	39 (35.5%)	60 (24.8%)	
ESKAPE	NO	80 (60.6%)	70 (63.6%)	150 (62.0%)	0.690
	YES	52 (39.4%)	40 (36.4%)	92 (38.0%)	
MDR	NO	112 (84.8%)	94 (85.5%)	206 (85.1%)	0.521
	YES	20 (15.2%)	16 (14.5%)	36 (14.9%)	

CCI, Charlson Comorbidity Index; ASA-PS, American Society of Anesthesiologists Physical Status; Indications, AAC, acute acalculosus cholecystitis; ACC, acute calculosus cholecystitis; EVF, empyema visicae felleae; HVF, hydrops vesicae felleae; GP, gallbladder perforation; ESKAPE, E, *Enterococcus faecium*; S, *Staphylococcus aureus*; K, *Klebsiella pneumoniae*; A, *Acinetobacter Baumannii*; P, *Pseudomonas aeruginosa*; E, *Enterobacter* spp.; MDR, multidrug resistance.

Microbiological samples were collected from 379 patients (sampling rate: 57.8%) and a pathogen was isolated in 248 patients (37.8%) (acute CCY: 136, 20.7%; PTGBD: 112, 17.1%). Furthermore, 133 patients (20.3%) had sterile AC (acute CCY: 100, 15.2%; PTGBD: 31, 4.7%).

Among the 248 patients with positive microbiological samples, 422 bacteria were isolated (acute CCY: 203, PTGBD: 219). Finally, 242 patients (acute CCY: 132, PTGBD: 110) with 412 positive results (acute CCY: 195, PTGBD: 217) were included in the statistical analysis (Figure 1).

### Distribution of bacteria by gram stain results

Gram-negative bacteria accounted for 60.9% (251/412) of the isolates. Table 2 shows the distribution of bacteria in the acute CCY and PTGBD groups.

**Table 2:** Distribution pathogens in patients with acute cholecystectomy or percutaneous transhepatic gallbladder drainage.

Pathogen	CCY (*n*, %)	PTGBD (*n*, %)	Total (*n*, %)
Gram-negative	**118 (60.5%)**	**133** **(61.3%)**	**251 (60.9%)**
*Escherichia spp.*	45 (23.1%)	61 (28.1%)	106 (25.7%)
*Klebsiella spp.*	36 (18.5%)	19 (8.8%)	55 (13.3%)
*Enterobacter* spp.	12 (6.2%)	19 (8.8%)	31 (7.5%)
*Proteus* spp.	4 (2.1%)	6 (2.8%)	10 (2.4%)
*Pseudomonas* spp.	4 (2.1%)	6 (2.8%)	10 (2.4%)
Other*	17 (8.7%)	22 (10.1%)	39 (9.5%)
Gram-positive	**77 (39.5%)**	**84** **(38.7%)**	**161** **(39.1%)**
*Streptococcus*	25 (12.8%)	32 (14.7%)	57 (13.8%)
*Enterococcus*	26 (13.3%)	30 (13.8%)	56 (13.6%)
*Clostridium*	6 (3.1%)	9 (4.1%)	15 (3.6%)
*Staphylococcus*	10 (5.1%)	1 (0.5%)	11 (2.7%)
Other**	10 (5.1%)	12 (5.5%)	22 (5.3%)
Total	**195** **(100%)**	**217** **(100%)**	**412** **(100%)**

ACC, acute cholecystectomy; PTGBD, percutaneous transhepatic gallbladder drainage.

Bold values indicate the main bacterial groups (Gram-negative and Gram-positive) and the total values.

* Bacteroides, Prevotella, Citrobacter, Serratia, Phocaeicola, Achromobacter, Acinetobacter, Aeromonas, Cronobacter, Fusobacterium, Haemophilus, Hafnia, Massilia, Morganella, Providencia.

** Peptostreptococcus, Cutibacterium, Gemella, Lactococcus, Parvimonas, Schaalia, Fannyhessea, Finegoldia, Granulicatella, Lacticaseibacillus, Levilactobacillus, Pediococcus, Priestia, Veillonella.

The most frequently isolated Gram negative and Gram positive bacteria were *E. coli* and *Streptococcus* spp in both groups, respectively (see Table 2).

### Incidence of ESKAPE pathogens

Overall, 109 of the 412 bacteria (26.5%) were ESKAPE pathogens (Table 3). The most common were *K. pneumoniae* (42, 10.2%) and Enterobacter cloacae (18, 4.4%).

**Table 3:** Distribution of ESKAPE pathogens.

ESKAPE	Genus	Species	Frequency	%
E	*Enterococcus*	*Faecium*	8	1.9
S	*Staphylococcus*	*Aureus*	5	1.2
K	*Klebsiella*	Aerogenes	4	1.0
		Oxytoca	8	1.9
		*Pneumoniae*	42	10.2
		Variicola	1	0.2
A	*Acinetobacter*	*Baumannii*	0	0.0
P	*Pseudomonas*	*Aeruginosa*	9	2.2
E	*Cronobacter*	Sakazakii	1	0.2
	*Enterobacter*	Asburiae	5	1.2
		Cloacae	18	4.4
		Kobei	2	0.5
		Ludwigii	6	1.5
Total			109	26.5

ESKAPE: E, *Enterococcus faecium*; S, *Staphylococcus aureus*; K, *Klebsiella pneumoniae*; A, *Acinetobacter baumannii*; P, *Pseudomonas aeruginosa*; E, *Enterobacter* spp.

*N* = 412, 100%.

### Cumulative antibiogram

Tables 4, 5 show the antibiotic sensitivity and MDR rates of the most common bacteria in AC samples.

**Table 4:** Cumulative antibiogram of gram-negative pathogens.

Group	N	AMP	AMC	CXM	CRO	CAZ	FEP	TZP	ETP	MEM	CIP	AMK	GEN	SXT	MDR (%)	MDR (*n*)	ESBL (%)
*Klebsiella* spp.	55	0	73	83	85	85	85	85	100	100	89	93	96	89	14.5	8	5.6
*Escherichia* coli	106	54	81	85	91	91	91	90	100	100	80	92	91	78	23.6	25	
*Enterobacter* spp.	13	0	0	33	100	100	100	100	100	100	100	100	100	100	23.1	3	
*Proteus* spp.	10	50	60	50	78	78	89	90	100	100	78	100	100	78	20.0	2	
*Enterobacter* cloacae	18	0	0	75	83	82	83	83	100	100	100	100	100	100	5.6	1	
*Pseudomonas* spp.	10	IR	IR	IR	IR	80	90	90	IR	90	60	100	80	IR	10.0	1	

IR, intrinsic resistance; AMP, ampicillin; AMC co-amoxiclav; CXM, cefuroxim; CTX, cefotaxim; CRO, ceftriaxon; CAZ, ceftazidim; FEP, cefepim; TZP, piperacillin/tazobactam; ETP, ertapenem; MEM, meropenem; CIP, ciprofloxacin; AMK, amikacin; GEN, gentamicin; SXT, sulfamethoxazole/trimethoprim; MDR, multidrug resistance; ESBL, Extended-Spectrum Beta-Lactamases producing Enterobacterales.

Green: Susceptibility rate over 90%, may be prescribed with empirical antibiotics even in severe infections. Yellow: Susceptibility rate over 80%, may be prescribed with empirical antibiotics except in severe infections. Red: Susceptibility rate below 80%, should not be prescribed with empirical antibiotics.

**Table 5:** Cumulative antibiogram of gram-positive pathogens.

Species	Susceptibility (%) to antimicrobial agents (each antibiotic expressed as %)																
	N	OXA	AMP	AMC	CXM	CC	CIP	ERY	GM	HLAR	LNZ	TEC	VA	TGC	SXT	MDR (%)	MDR
*Clostridium* spp	15	IR	ND	100.0	IR	100.0	IR	IR	IR	IR	ND	ND	ND	ND	ND	0	0
*Enterococcus* faecalis	41	IR	100.0	100.0	IR	IR	92.9	IR	ND	75.8	100	100	100	100	100	0	0
*Enterococcus* spp	15	IR	60.0	64.3	IR	IR	100.0	IR	ND	80	100	93.3	85.7	100	ND	20	3
*Staphylococcus aureus*	5	100	100.0	100.0	100.0	80.0	100.0	80.0	100	ND	ND	100	100	100	100.0	0	0
*Streptococcus* spp	56	IR	100.0	100.0	100.0	86.5	100.0	85.7	ND	ND	ND	ND	100	ND	50	0	0

IR, intrinsic resistance; OXA, oxacillin; AMP, ampicillin; AMC, co-amoxiclav; CXM, cefuroxime; CC, clindamycin; CIP, ciproﬂoxacin; ERY, Erythromycin; GM, gentamicin; HLAR, high-level aminoglycoside resistance; LNZ, linezolid; TEC, teicoplanin; VA, vancomycin; TGC, tigecyclin; SXT, sulfamethoxazole/trimethoprim; MDR, multidrug resistance; ND, no data; it has not been studied) From Staphylococcus spp. only pathogen *S. aureus* was part of our investigation.

Green: Susceptibility rate over 90%, may be prescribed with empirical antibiotics even in severe infections. Yellow: Susceptibility rate over 80%, may be prescribed with empirical antibiotics except in severe infections. Red: Susceptibility rate below 80%, should not be prescribed with empirical antibiotics.

The susceptibility of *E. coli* to third- and fourth -generation cephalosporins and carbapenems were over 90%, but decreased susceptibility was observed against ciprofloxacin (80%) and sulfamethoxazole–trimethoprim (78%); and almost 25% of the isolates were MDR pathogens. The susceptibility ratio of *Klebsiella spp.* was less than 90% for all beta-lactams (except for carbapenems); and 14.5% (*n* = 8) of the strains were MDR pathogens. Additionally, their susceptibility to ciprofloxacin was also low (89%). Meanwhile, the ratio of ESBL-producing *Enterobacterales* was 5.6% (*n* = 23).

A cumulative antibiogram was created for Gram-positive bacteria, too. *Enterococcus* spp. showed a susceptibility of 60% to ampicillin, and 64.3% to amoxicillin. Altogether 14.3% of *Enterococcus spp*. were resistant to vancomycin. Besides quinolones, adequate susceptibility was observed against reserve antibiotics (linezolid, tigecycline) (15). From Staphylococcus spp. only pathogen S. aureus was part of our investigation. Notably, methicillin-resistant *S. aureus* (MRSA) was not isolated from bile during the study period.

**Figure 2:**
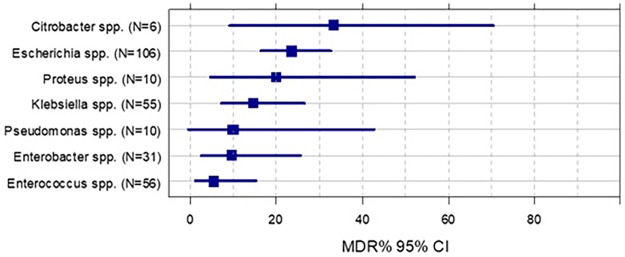
Proportion of multidrug-resistant (MDR) bacteria among different bacterial species.

Regarding MDR pathogens, the highest MDR rate was observed in *Citrobacter* spp. strains, followed by *Escherichia* spp., *Proteus*s and *Klebsiella*. Among Gram-positive bacteria, only *Enterococcus* spp. had MDR strains (<10%), all of them were *E. faecium* strains (Figure 2).

### Grade vs. ESKAPE and MDR

Table 6 shows the presence of ESKAPE and MDR bacteria stratified by AC grade. A higher grade was associated with a higher incidence of ESKAPE pathogens: in cases of grade 1 AC, 30.2%, in grade 2 38.9% of bacteria were ESKAPE pathogens, which increased to 46.7% in grade 3 inflammations (*p* = 0.304). Additionally, the higher the grade of inflammation, the higher was the incidence of MDR pathogens, although the difference was not statistically significant (*p* = 0.230).

**Table 6:** Presence of ESKAPE and MDR pathogens according to the severity of acute cholecystitis.

Pathogen category		Grade 1 (*n*, %)	Grade 2 (*n*, %)	Grade 3 (*n*, %)	NA	Total (*n*, %)	*p*-value
ESKAPE	NO	37 (69.8%)	96 (61.1%)	16 (53.3%)	1	149 (62%)	0.304
	YES	16 (30.2%)	61 (38.9%)	14 (46.7%)	1	91 (38%)	
MDR	NO	48 (90.6%)	133 (84.7%)	23 (76.7%)		206 (85.1%)	0.230
	YES	5 (9.4%)	24 (15.3%)	7 (23.3%)		36 (14.9%)	

ESKAPE: E, *Enterococcus faecium*; S, *Staphylococcus aureus*; K, *Klebsiella pneumoniae*; A, *Acinetobacter baumannii*; P, *Pseudomonas aeruginosa*; E, *Enterobacter* spp.; MDR, multidrug resistance.

### Time frame vs. ESKAPE and MDR

Table 7 shows the interval between the onset of symptoms and hospitalization. Beyond 72 h after the beginning of the symptoms we detected higher ratio of Gram-negative bacteria (55%) than within 72 h (43.7%; *p* = 0.053). The incidence of ESKAPE and MDR pathogens did not change as the time frame increased.

**Table 7:** Evaluation of time frame and gram-negative, gram-positive, ESKAPE, MDR, and certain bacteria in acute cholecystitis.

Bacterial category		Within 72 h (*n*, %)	After 72 h (*n*, %)	NA	Total (*n*, %)	*p*-value
Gram-positive		45 (34.1%)	15 (18.8%)	7	67 (27.7%)	0.053
Gram-negative		57 (43.2%)	44 (55%)	14	115 (47.5%)	
Gram-neg. AND pos.		30 (22.7%)	21 (26.3%)	9	60 (24.8%)	
ESKAPE	NO	79 (59.85%)	53 (66.25%)	18	132 (54.54%)	0.3829
	YES	53 (40.15%)	27 (33.75%)	19	73 (30.16%)	
MDR	NO	112 (84.8%)	67 (83.8%)	27	206 (85.1%)	0.847
	YES	20 (15.2%)	13 (16.3%)	3	36 (14.9%)	

ESKAPE: E, *Enterococcus faecium*; S, *Staphylococcus aureus*; K, *Klebsiella pneumoniae*; A, *Acinetobacter baumannii*; P, *Pseudomonas aeruginosa*; E, *Enterobacter* spp.; MDR, multidrug resistance.

## Discussion

The objective of this study was to analyze the microbiological spectrum of bile cultures in patients with acute cholecystitis, with particular emphasis on ESKAPE and multidrug-resistant pathogens, and to evaluate their antibiotic susceptibility patterns. This study represents the first comprehensive analysis from Central Europe describing bile culture results and cumulative antibiograms in acute cholecystitis.

Empirical antibiotic therapy should be initiated at the time of AC diagnosis, with subsequent potential adjustment to targeted therapy based on microbiological sampling and the local antibiogram (7). In our department, the average sampling ratio in AC was 58% and showed a constantly increasing tendency from 2005 to 2019 (from 13% to 78.2%). Both the TG13 and TG18 guidelines recommend microbiological sampling in all AC cases (7, 16).

Gram-negative bacteria, which are present in the gut, usually cannot be isolated in the bile of healthy individuals. In mild AC, the bile can be sterile, but it is colonized by pathogens and non-pathogenic bacteria as AC progresses (6). According to the literature in patients who have undergone elective laparoscopic cholecystectomy, bile was colonized by pathogens in 9%–42% of cases. This ratio is considerably higher in cases of AC and may reach 35%–65% (17, 18). In our tertiary center, the positivity rate of samples was high (65.43%), which may be attributed to the fact that several severe cases (Grade 2 AC ratio was 65.4%; Grade 3 AC ratio was 12.5%) were seen at our tertiary center.

We observed a predominance of Gram-negative bacteria: among these, members of the *Enterobacterales* family was the predominant strain. Similar to the study of Nitzan et al. from 2017, *E. coli* was the most commonly isolated strain in our study (19). Enterobacterales family use AcrAB efflux pump, which is found in Escherichia coli, Salmonella, Shigella, Klebsiella, and other pathogens, to resist both bile salts (20) and antibiotics (some ß-lactams: ampicillin, piperacillin; cephalosporins: cefepime, cefotaxime; fluoroquinolones: ciprofloxacin, levofloxacin, macrolides, tetracyclines) (21, 22), making it possible for these bacteria to survive under extreme environmental conditions (23), therefore, colonizes the bile more easily, resulting in inflammation (6, 24–25).

As recommended in TG18, empirical antibiotic therapy, guided by the local cumulative antibiogram, should be administered to patients with AC of all severity grades (7). TG18 recommends empiric therapy for resistant isolates if they occur in more than 20% of patients (7, 26). Considering the usual cut-off values an antibiotic can be used as empirical treatment in severe infections if the cumulative susceptibility rate is over 90% (50). If the susceptibility rate is over 80% the agent can be used as empirical treatment except for severe infections. If susceptibility rate is below 80%, the antibiotic agent is not recommended as empirical therapy (27).

In mild AC cases (as per TG18 grade 1) it is unclear if bacteria play a significant role in AC; therefore, in these patients the aim of antibiotic therapy is prophylactic, preventing the progression of infection (7). According to our results, the first choice for empirical antimicrobial therapy in grade 1 AC should be cefuroxime as more than 80% of isolates were susceptible to this agent. Ceftriaxone and ceftazidime are also effective alternatives, with susceptibility rates of 85% for both agents. In contrast *Escherichia coli* was susceptible only in 54% to ampicillin and *Klebsiella* spp. has shown resistance to penicillin combinations with *β*-lactamase inhibitors e.g., amoxicillin-clavulanic acid (only 73% was susceptible) and moderate susceptibility to cefuroxime (83%), ceftriaxone (85%), and ceftazidime (85%). Based on these findings, cefuroxime should be the primary empirical choice, followed by ceftriaxone, with ceftazidime as an additional alternative if needed. In cases of severe infections (Grade 2 and 3), third- and fourth generation cephalosporins, piperacillin–tazobactam or carbapenems should be used because *Escherichia* spp., *Klebsiella* spp., and *Enterobacter* spp. are the most likely pathogens, which were ≥90% sensitive to these antibiotics. An exception to the above is *Proteus* spp., which were only 78% sensitive to ceftriaxone and ceftazidime. In grade 3 cases, where the prevalence of ESKAPE and multidrug-resistant pathogens was the highest (28.6% ESKAPE and 14.3% MDR—not shown in tables), treatment should prioritize third generation cephalosporin, carbapenems or cefepime to ensure adequate coverage.

Based on the local antibiogram, our recommended empirical antimicrobial therapy matches those in TG18. TG18 recommends considering the grade of AC when choosing the appropriate empirical antibiotic: piperacillin–tazobactam or third- and fourth-generation cephalosporins should be used for Grade 2 AC infections and piperacillin-tazobactam, cefepime, ceftazidime or carbapenems (imipenem/cilastatin, meropenem) should be used in grade 3 AC (7). According to TG18, agents effective against anaerobes (e.g., metronidazole, tinidazole, clindamycin) are recommended if the patient has undergone biliary–enteric anastomosis; in such cases, third- and fourth-generation cephalosporins combined with metronidazole are recommended (7). In our study, *Clostridium* spp. was isolated in 3.6% of the samples, whereas the ratio of other anaerobes (*Bacteroides* spp., *Prevotella* spp., *Fusobacterium* spp.) were negligible. Based on our results, empirical coverage for anaerobes is not mandatory.

The decreased susceptibility of *E. coli*, *Proteus* spp., and *Pseudomonas* spp. to ciprofloxacin should be noted; the probable reason for this is that fluoroquinolones are widely used for several conditions in Hungary (28, 29).

In this study, the prevalence of ESBL-producing strains was 5.6%. However, the distribution of ESBL-producing pathogens differs widely from region to region. For example, in two German university hospitals the rate of ESBL-producing *E. coli* in acute cholangitis was 35.1% (30). In an Italian multicenter study, intra-abdominal infections owing to AC were investigated. In healthcare-associated infections, ESBL-producing strains included one case of *E. coli* and one case of *K. pneumoniae*. In contrast, in community-acquired infections, 11 out of 85 *E. coli* isolates (12.94%) and 3 out of 16 *K. pneumoniae* isolates (18.75%) were ESBL-producing strains (31).

Regarding Gram-positive strains, members of *Enterococcus* spp. were susceptible to *β*-lactam antibiotics were only found in 60%–65% in our study. Based on the literature, community acquired *E. faecium* strains may be ampicillin-resistant in up to 23% of cases (32). Within the genus, the ratio of vancomycin-resistant *Enterococci* was 14.3%, which was close to the European value resulted from invasive samples in the 2021 EARS-NET Report (17.2%) (33).

*S. aureus* infection is rare in AC; it was isolated in only 0.8%–5.6% of samples in previous studies (34–36). In our study, *Staphylococcus* spp. (*n* = 11) was isolated in 2.67% of samples, but from this group only *Staphylococcus aureus* is a pathogen (*n* = 5; 1.21%). MRSA (Methicillin-resistant *Staphylococcus aureus*) was not isolated. In their study, Merchant and Falsey isolated *S. aureus* in the bile of three patients with AC, and MRSA was isolated in two of these cases. In both cases, intravenous catheter infection was determined as the origin of infection (37).

The incidence of ESKAPE and MDR pathogens according to the grade (1–3) of AC has not been explored before. According to the literature in cases where ESKAPE pathogens were isolated, inappropriate empirical antibiotic treatment was associated with significantly higher mortality among patients with sepsis (38–42). As a second objective, we have established that the higher the grade of inflammation, the higher the incidence of ESKAPE pathogens. Based on our results, an ESKAPE pathogen was isolated in 30.2% of the cases of AC with grade 1, 38.9% of the cases of AC with grade 2 while in 46.7% in grade 3. The difference was not significant (*p* = 0.304) The difference was not significant (*p* = 0.304), however, an increasing trend was observed with the progression of inflammation severity. There was also an increasing tendency in the rate of MDR pathogens as the grade was more severe, while the rate of MDR pathogens was 9.4% in grade 1, 15.3% in grade 2 and in grade 3 it was 23.3%.

It should be noted, however, that in cases of a mild AC with grade 1 inflammation, the main problem is not bacterial infection as drainage of the gallbladder is most commonly obstructed by a gallstone, which increases pressure in the gallbladder, causing oedema and local inflammation in its wall (43). In such cases, early CCY and one-shot perioperative prophylaxis with cefuroxime are sufficient. In cases of grades 2 and 3 AC the appropriate empirical antibiotic treatment and later the targeted therapy based on susceptibility test(s) have a major role because of the increased presence of ESKAPE pathogens. In moderate or severe AC (grade 2 or 3) cases, the use of antibiotics is therapeutic and should be continued at least until CCY is performed, there is resolution of symptoms, or laboratory values have been improved (44).

The relationship between the time frame, the presence of ESKAPE, and MDR strains has not been investigated yet. We found no correlation between ESKAPE pathogens, MDR strains, and the time frame.

This study has some limitations. Since data were obtained retrospectively from electronic patient records, patient selection was based on ICD-10 codes diagnosis. As a single-center study, its findings may not be generalizable to other institutions with differing patient populations and antibiotic resistance patterns. Unfortunately, we detected that the number of blood cultures collected during the study period were low, therefore we did not include them in our investigation.

The present study provides a summary about the bacteriology of AC in a Hungarian tertiary-care surgical unit. Based on the results we could make more specific recommendations for empirical antibiotic therapy in our hospital based on the local antibiogram. A 100% sampling ratio in both acute CCY and PTGBD should be aimed. Local continuous monitoring of cumulative antibiogram provides safe and appropriate therapy for patients with acute cholecystitis (45). In higher grade of AC the rate of ESKAPE pathogens show increasing tendency so early adequate empirical antibiotic treatment is indispensable in the management of AC.

## Conclusion

This study determines the microbiological profile of bile cultures in patients with acute cholecystitis, including the presence of ESKAPE and multidrug-resistant (MDR) pathogens. However, the detection of these pathogens remains clinically relevant, as it influences empirical antibiotic selection and treatment outcomes.

Given the findings, empirical antibiotic therapy should be adjusted according to AC severity. Cefuroxime remains a suitable first-line agent for mild AC, while third-generation cephalosporins, piperacillin-tazobactam should be used for moderate AC. In cases of severe AC, carbapenems or cefepime should be prioritized to ensure adequate coverage of MDR and ESKAPE pathogens. Routine bile sampling should be prioritized in Grade II/III AC to guide targeted therapy and reduce inappropriate antibiotic use.

By integrating these findings into clinical practice, more effective antimicrobial strategies can be implemented, ultimately improving patient outcomes and minimizing the burden of antimicrobial resistance. Future research should include prospective studies with systematic bile sampling and outcome-based analyses to better define the clinical impact of pathogen-specific antibiotic therapy in acute cholecystitis.

## Data Availability

The original contributions presented in the study are included in the article. Further inquiries can be directed to the corresponding author.
